# Resistance Against Cassava Brown Streak Viruses From Africa in Cassava Germplasm From South America

**DOI:** 10.3389/fpls.2019.00567

**Published:** 2019-05-10

**Authors:** Samar Sheat, Bettina Fuerholzner, Beate Stein, Stephan Winter

**Affiliations:** Plant Virus Department, Leibniz Institute DSMZ-German Collection of Microorganisms and Cell Cultures, Braunschweig, Germany

**Keywords:** cassava brown streak virus, cassava brown streak disease, virus resistance, South American cassava, cassava diversity

## Abstract

Cassava brown streak disease (CBSD) is a severe virus disease of cassava and prevalent in the eastern regions of Africa. The disease is characterized by distinct vein chlorosis and streak symptoms on leaves and stems and necrosis of storage roots. This necrosis can encompass large areas of the root, rendering it inedible so that the entire cassava harvest can be lost. African cassava varieties are susceptible to either of the two viruses causing the disease, cassava brown streak virus (CBSV) and Uganda cassava brown streak virus, and while there are less sensitive varieties, all cassava eventually succumb to the disease. The lack of CBSD resistance in African cassava varieties prompted this search for new sources of virus resistance in the diversity of South American cassava germplasm held in the collection at International Center for Tropical Agriculture, Columbia. Our search for CBSD resistance in South American cassava germplasm accessions revealed that most of the 238 South American cassava lines infected with CBSV established systemic virus infections with moderate to severe disease symptoms on leaves and stems. Fifteen cassava accessions did not become virus infected, remained free of symptoms, and CBSV was undetected by qRT-PCR. When tuberous roots of those lines were examined, necrotic tissue was found in eight lines and CBSV was detected. The remaining seven cassava accessions remained clear of symptoms on all tissues and organs and were virus free. A broad spectrum of virus resistance also including other virus isolates was confirmed for the breeding lines DSC167 and DSC118. While detailed infection experiments with other cassava lines selected for resistance are still ongoing, this indicates that the resistance identified may also hold against a broader diversity of CBSVs. Taken together, we present the results of a comprehensive study on CBSV resistance and susceptibility in cassava germplasm accessions from South America. The virus resistance in cassava germplasm identified provides compelling evidence for the invaluable contribution of germplasm collections to supply the genetic resources for the improvement of our crops.

## Introduction

Cassava brown streak disease (CBSD), the most serious threat to cassava cultivation in East and Central Africa, is caused by two virus species, *cassava brown streak virus* (CBSV) and *Ugandan cassava brown streak virus* (UCBSV; Winter et al., 2010), both members of the genus *Ipomovirus* in the family Potyviridae (ICTV online)^1^. UCBSV and CBSV (U/CBSV) while genetically distinct, cause similar symptoms in leaves, stems, and root tissues of cassava (Hillocks and Jennings, 2003; Winter et al., 2010; Hillocks et al., 2016), including leaf chlorosis, brown streaks on stems, and brown corky necrosis on storage roots (Patil et al., 2015). Despite the aboveground symptoms, appearance of the plant can be vigorous because it is the necrosis on tuberous storage roots that renders parts or the entire root inedible (Kaweesi et al., 2014; Hillocks et al., 2016). The impact from CBSD is devastating (Legg et al., 2014; Patil et al., 2015) in the regions of Eastern and Central Africa, where the disease is now established and from where it is spreading toward the neighboring countries.

Intensive breeding efforts to enhance resistance against U/CBSV have resulted in varieties that although being virus infected show only mild symptoms on leaves and stems and much fewer root necrosis symptoms (Jennings, 1957; Kanju et al., 2012; Kaweesi et al., 2014; Kawuki et al., 2016; Masinde et al., 2018; Mukiibi et al., 2019). However, despite of progress made, cassava varieties with high resistance against CBSD are not yet found (Abaca et al., 2013; Bart and Taylor, 2017). All African cassava cultivars can become infected with U/CBSV (Winter et al., 2010; Kaweesi et al., 2014; Maruthi et al., 2014) and eventually exhibit more or less pronounced necrosis symptoms on roots. Because of the limited scope to find natural sources of U/CBSV resistance in African cassava varieties, we expanded our search to South America, the center of diversity of the crop. The genetic diversity of South American cassava is preserved in approximately 6,400 cassava germplasm accessions, 5,477 of which are kept in field and *in vitro* collections at the International Center for Tropical Agriculture (CIAT), Cali, Colombia. Around 630 accessions of this collection represent its core diversity (Hershey et al., 1994).

The underlying question of this virus study was whether resistance against a virus from Africa can be found in cassava germplasm from South America, where the virus does not exist. To address this, we developed a highly efficient virus inoculation and evaluation scheme and used a severe isolate of CBSV to infect 238 cassava lines originating from South America and an additional 42 cassava varieties from Africa. In this article, we report the results from our virus study and present South American cassava varieties with resistance against CBSV. The South American cassava genotypes we identified either did not become virus infected at all or restricted virus infections to root tissues only. While comprehensive infection studies with other U/CBSV isolates are still ongoing, results from infection studies with a limited number of cassava lines already indicate that resistance is encompassing a broad range of viruses causing CBSD.

## Materials and Methods

### Cassava Varieties, Breeding Lines, and Land Races

The South American cassava germplasm lines (238) were obtained as tissue cultured materials from the CIAT germplasm collection^2^. Cassava varieties, land races, and breeding lines from Africa were either from the DSMZ Plant Virus Collection or provided by Prof. Maruthi (NRI, United Kingdom). The tissue cultured plants were propagated *in vitro*, hardened, and subsequently established in the glasshouse and grown under ambient conditions at 26 to 30°C with additional light provided.

### Viruses, Virus Isolates, and Maintenance

For all virus experiments, the CBSV reference isolates CBSV-Mo83 (DSMZ PV-0949, FN434436), CBSV-Ug65 (DSMZ PV-0996), and CBSV-Tan70 (DSMZ PV-0957, FN 434437) and the UCBSV reference isolate UCBSV-Ke125 (DSMZ PV-0912, FN433930) were used. The viruses were maintained in the cassava varieties TME7 or TMS-96/0304 that were propagated through stem cuttings to produce sufficient amounts of axillary buds for grafting.

### Plant Infections

Bud grafting similar to the method described by Wagaba et al. (2013) was used for high-throughput screening. Axillary buds were taken from virus-infected cassava, inserted into the cassava line to be tested, and protected from desiccation by a layer of parafilm for a period of 10 days. To increase the inoculation pressure and, thus, the chances for virus transmission, two axillary buds from virus-infected cassava were inserted into each of the rootstocks. At least two to three plants from each accession were included in the bud grafting experiments and subsequently kept for approximately 14 days in a foil tunnel at high humidity to protect from withering. Thereafter, plants were grown in the glasshouse inspected daily for virus symptoms.

### Pathogenicity of Virus Isolates

Prior to virus screening, the pathogenicity of virus isolates was assessed by comparing symptom severity and virus titer to then use the most aggressive virus isolate for a first, large-scale virus screening. For the bioassay, five plants of two African varieties – the variety “Albert,” which responds with severe symptoms to U/CBSV infections (sensitive), and the resistant variety “Namikonga,” responding to virus infections with limited symptom expression (tolerant) (Kaweesi et al., 2014; Maruthi et al., 2014) – were infected with the reference virus isolates. Axillary buds from virus-infected plants were grafted onto 2- to 3-month-old plants from each variety, which were then monitored for symptom development and virus accumulation. For virus analysis, three individual leaves (top, middle, and basal leaf) from each virus-infected plant were collected 10 days after grafting (dag) and then at 10-day intervals for 60 days. Virus concentration was then determined, for each sample separately, using quantitative reverse transcription PCR (qRT-PCR).

### Symptoms Assessment

Symptoms on leaves were assessed using a severity scale ranging from 1, for leaves without symptoms, to 5, for severe symptoms on leaves (Rwegasira and Rey, 2012). The mean symptom severity was then calculated in a score between 2 and 5.

For large-scale virus screening, symptoms on cassava leaves and stems were evaluated using a severity record S that was defined for the purpose: S0, no symptoms on leaves and stems; S?, inconspicuous symptoms on leaves and stems; S+, moderate symptoms on leaves only; S++, severe symptoms on leaves and stems; and S+++, wilting of the stem followed by plant death.

### Screening for Virus Resistance

**FIGURE 1:**
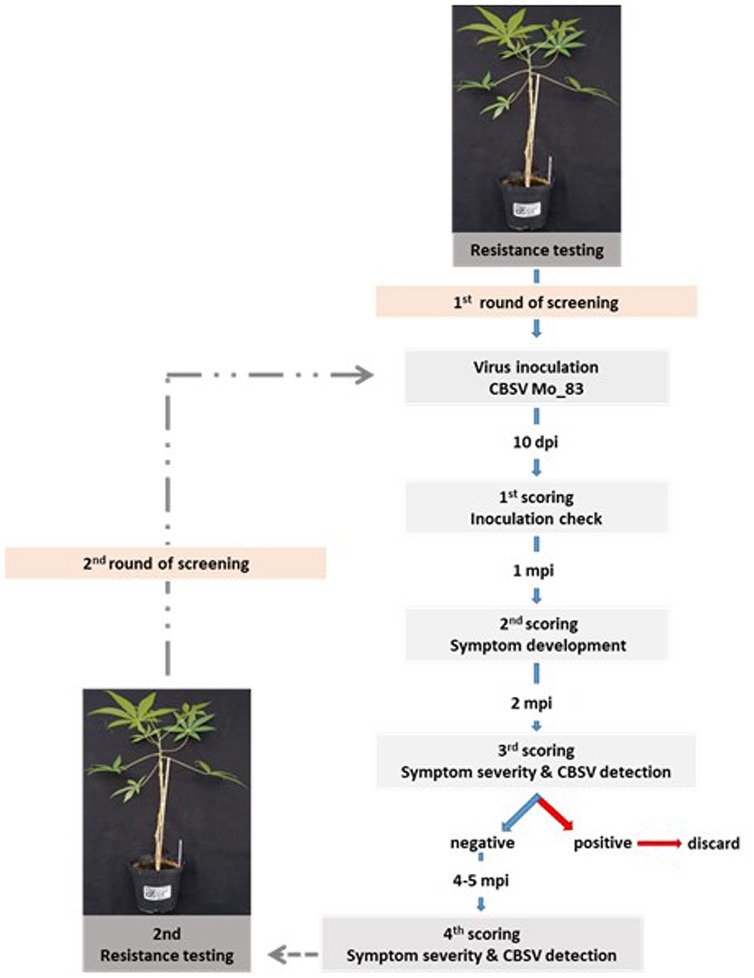
Screening strategy to test for virus resistance in cassava germplasm. Cassava lines that did not become virus infected during the first virus screening were subjected to a second round of virus testing to confirm their resistance status. Each screening phase was for 8 months and included symptom assessment and virus testing by quantitative reverse transcription PCR (qRT-PCR).

For the initial screening, the virus isolate CBSV-Mo83 was used to infect cassava. Plantlets established from tissue culture (two to three plants/accession) were infected by bud grafting, checked for the infection status at 10 dag, and inspected for symptom development. Cassava plants that remained symptomless for 1 month were decapitated by removing the apical parts of the shoots to force flushing of axillary buds. Viruses were monitored for a further 4 to 6 weeks. All cassava plants subjected to bud grafting, including those highly susceptible plants that wilted and died within the first 2 to 3 weeks after grafting, were tested for virus presence by qRT-PCR. Virus symptoms and detection of CBSV by qRT-PCR were taken as proof for susceptibility of a cassava accession, and even if only one plant became infected, the line was discarded and exempt from further testing. Monitoring and virus testing continued with symptomless and qRT-PCR-negative plants for a further 5–8 months, after which cassava lines that remained free of symptoms and virus were subjected to a second round of virus screening using five plants per cassava line (Figure 1).

### Evaluation of Virus Movement and Replication

To study virus movement in the cassava accession DSC167 that was found highly resistant against CBSV, scions (5 cm, 2 knots) from this line were side grafted (Winter et al., 2010) onto healthy cassava TMS-96/0304 rootstocks. Similarly, scions from the susceptible variety Albert were side grafted onto TMS-96/0304 to serve as infection control. After establishment, sprouting of the side-grafted scions was encouraged by removing the leaves of the rootstock just below the graft insertion and the apical portions of the rootstock. Once branches had formed, axillary buds from CBSV-Mo83-infected cassava were graft inserted into the TMS-96/0304 rootstocks to infect the branched TMS-96/0304/DSC167 and the TMS-96/0304/Albert chimeric plants, respectively. For this experiment, 12 TMS-96/0304/DSC167 and 6 TMS-96/0304/Albert plants were used.

### Virus Detection and Quantification

RNA from cassava was extracted using an RNA extraction kit following the manufacturer’s protocol (Epoch, United States). The integrity of the RNA was analyzed by gel electrophoresis, and RNA was quantified in a Qubit^®^fluorometer (Thermo Fisher Scientific, United States) using the Qubit RNA BR Assay Kit (Thermo Fisher Scientific, United States). Virus analysis by qRT-PCR was carried out by either an ep realplex4 (Eppendorf, Germany) Mastercycler equipped with the realplex4 software or a qTOWER^3^ (Analytik Jena, Germany) equipped with the qPCRsoft evaluation software.

For virus quantification in the varieties Albert and Namikonga, a SYBR Green qRT-PCR assay was performed using published primer sets (Adams et al., 2013) and cassava 4.1_010236 (acyl co-binding A) as an internal control for relative virus quantification (Hu et al., 2016; Supplementary Table 1).

One microliter of total RNA (20 ng/μl) was converted into cDNA in a reaction mixture containing 5 μl of Moloney Murine Leukemia Virus Reverse Transcriptase (M-MLV RT) buffer (2×), 0.25 μl of dNTPs, 0.3 μM oligo(dt), 0.3 μM random hexamer primers, 0.2 μl of M-MLV RT, and nuclease-free sterile water to reach a reaction volume of 25 μl. cDNA synthesis was done for 30 min at 43°C and diluted fivefold prior to SYBR Green Kapa (PEQLAB, Germany) qPCR. Reaction mixtures contained 10 μl of SYBR Master Mix, 0.15 μM CBSV primers (0.1 μM for UCBSV and for cassava 4.1_010236 primers), 5 μl of diluted cDNA template, and nuclease-free sterile water to a total reaction volume of 20 μl. Control reactions, non-template water, RNA from healthy cassava, and RNA from UCBSV-infected cassava were included in every reaction series to conduct qRT-PCR with two technical replicates. After an initial denaturation step for 3 min at 95°C, each two-step cycle consisted of denaturation for 10 s at 95°C and synthesis for 30 s at 60°C. PCR was done for 36 cycles followed by melting curve analysis to assess the specificity of the amplification.

To confirm presence/absence of U/CBSV in cassava, a one-step TaqMan assay was conducted (TaqMan Kit Maxima Probe/ROX qPCR Master Mix, Thermo Fisher Scientific, Germany) with virus-specific primers and probes and COX (cytochrome oxidase) as an internal control (Kaweesi et al., 2014; Supplementary Table 1). Reaction mixes for qRT-PCR contained 12.5 μl of Maxima Probe qPCR Master Mix (2×), 0.3 μM CBSV primers, and probe CBSV primers (0.4 μM for COX primers and probe, 0.4 μl UCBSV primers and probes), 5 μl of template, 0.15 μl of M-MLV RT, and nuclease-free sterile water to a total reaction volume of 25 μl. Each RNA was analyzed in two qRT-PCRs, and controls were included in every series. One-step qRT-PCRs were incubated for 30 min at 43°C for cDNA synthesis followed by an initial denaturation step for 2 min at 95°C and 40 cycles of denaturation (15 s at 95°C), annealing (30 s at 60°C), and synthesis (30 s at 72°C).

Cycle threshold (CT) values were used to calculate U/CBSV expression using the 2^−ΔΔCt^ method (Livak and Schmittgen, 2001). Virus expression values were estimated relative to CBSV-Mo83 concentrations determined in the uppermost youngest leaf. For variety, Albert recordings at 10 dag were taken for reference, while for the variety Namikonga, qRT-PCR reference measurements were taken 20 days after infection (dag) because of the delayed infection. To quantify virus in the South American cassava germplasm, expression values were calculated relative to CBSV-Mo83 concentrations recorded in the sixth uppermost leaf of the variety TMS-96/0304.

## Results

A highly efficient screening and evaluation protocol was prerequisite to test such large numbers of plants for virus infection responses and to identify virus resistance in individual plants. Prior to the virus study, UCBSV isolates representing the core diversity of virus genomes (Winter et al., 2010; Ndunguru et al., 2015; Mbewe et al., 2017) and having distinct biological features were compared to determine the most aggressive virus.

### CBSV-Mo83 Is the Most Pathogenic Virus Causing CBSD in Cassava

**FIGURE 2:**
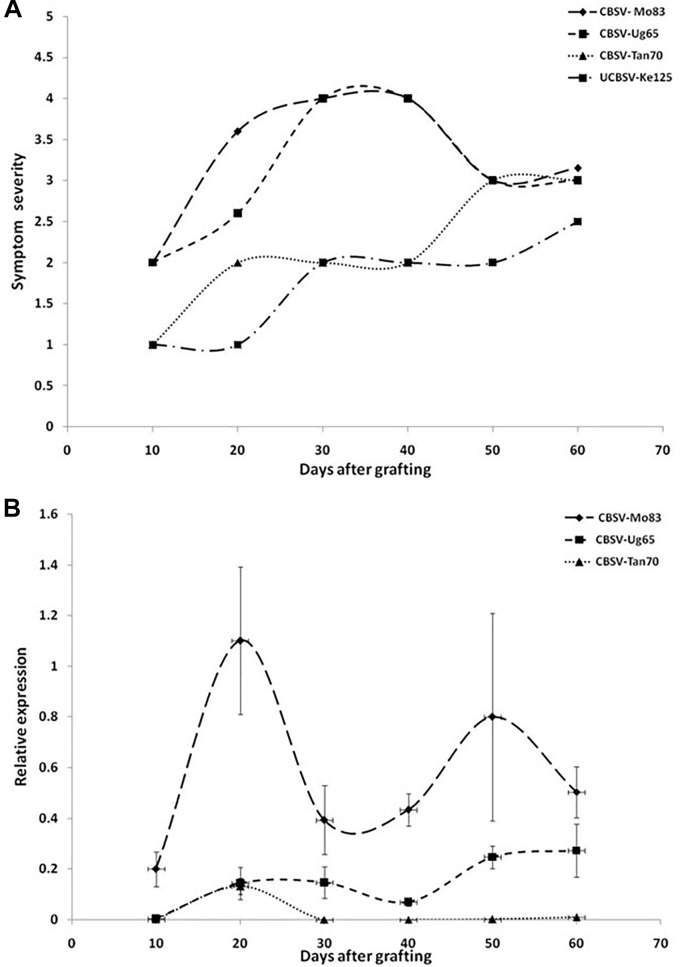
**(A)** Onset and severity of symptoms on leaves of five plants (*n* = 5) of the cassava variety Albert after infection with Uganda cassava brown streak virus (UCBSV) isolates. Symptoms became visible for infections with CBSV-Mo83 10 dag, but symptom development was delayed and less severe in infections with other virus isolates. **(B)** Accumulation of virus RNA during the infection process. Data obtained from qRT-PCR were normalized against acyl cobinding A to estimate virus expression (*y* axis) relative to CBSV-Mo83 values reached 10 dag in the uppermost youngest leaf. Error bars represent standard error of the mean (*n* = 5). Virus accumulation was rapid with CBSV-Mo83 and fluctuating over time with high amplitudes.

The infection processes of U/CBSV reference isolates were followed in the cassava varieties Albert (highly susceptible, sensitive) and Namikonga (resistant, tolerant) for 60 dag by evaluating the onset and severity of symptoms and quantifying virus load in leaves. In the variety Albert, symptoms of CBSV infections became visible already 10 dag, while symptoms of the UCBSV-Ke125 appeared much later and were generally mild and sometimes only barely visible during the first 30 dag (Figure 2A).

**FIGURE 3:**
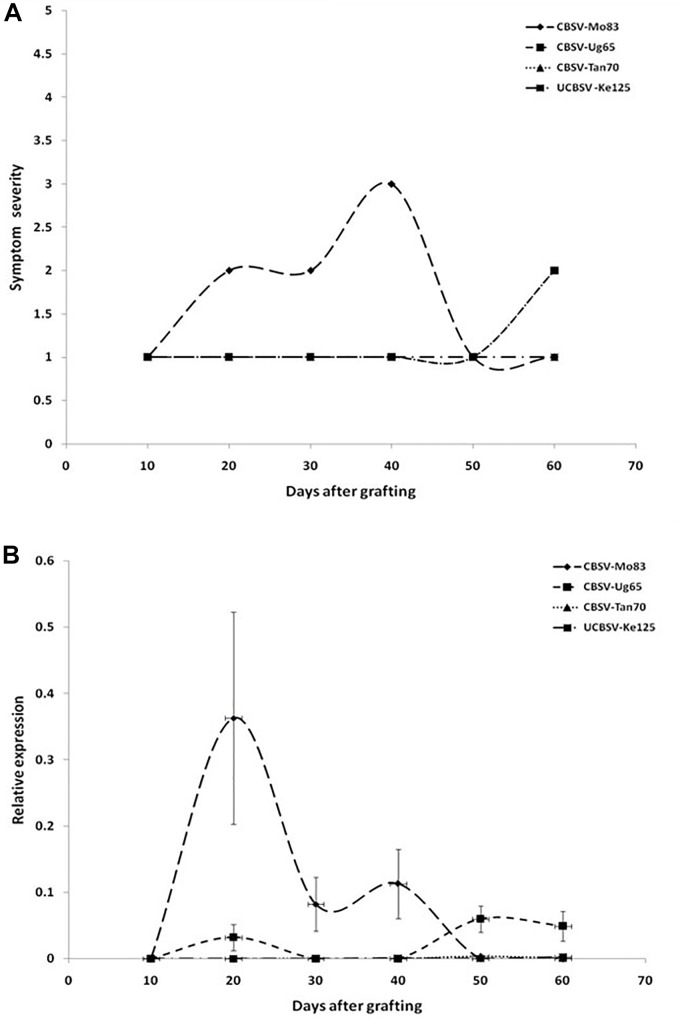
**(A)** Onset and severity of symptoms on leaves of five plants (*n* = 5) of the cassava variety Namikonga after infection with UCBSV isolates. Symptoms became noticeable in CBSV-Mo83 infections 20 dag, while symptoms of infections with other virus isolates were only barely noticeable. **(B)** Accumulation of virus RNA during the infection process. Data obtained from qRT-PCR were normalized against acyl cobinding A to estimate virus expression (*y* axis) relative to CBSV-Mo83 values reached 20 dag in the uppermost youngest leaf. Error bars represent standard error of the mean (*n* = 5). Virus accumulation was rapid with CBSV-Mo83 and fluctuating over time, while accumulation of other viruses was not remarkable.

All viruses caused streak symptoms on stems, which became evident on both varieties 60 dag, however, were most severe in CBSV-Mo83 infections. Virus concentrations in leaves fluctuated over time (Figure 2B), with higher amplitudes observed in CBSV-Mo83 infections. In the resistant variety Namikonga, very mild symptoms of CBSV-Mo83 infections became noticeable 20 dag, while symptoms caused by other virus isolates became visible only 50 dag. Only CBSV-Mo83 was readily detected in qRT-PCR assays, while all other viruses were only hardly detectable by qRT-PCR through the observation time (Figure 3B).

**FIGURE 4:**
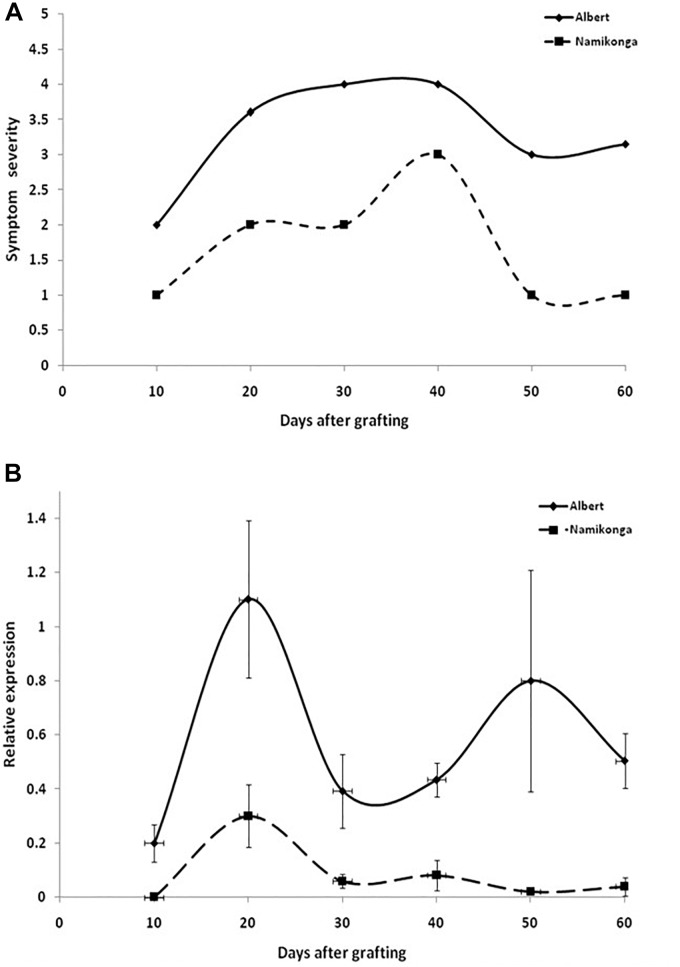
**(A)** Appearance and severity of symptoms on leaves of the cassava varieties Albert and Namikonga after infection with CBSV-Mo83. Symptoms became visible in both varieties after 10 dag but remained mild and less pronounced in Namikonga. **(B)** Accumulation of virus RNA during the infection process. Data obtained from qRT-PCR were normalized against acyl cobinding A to estimate virus expression (*y* axis) relative to CBSV-Mo83 values reached 10 dag in the uppermost youngest leaf. At this time point, there was no detectable virus in Namikonga. Error bars represent standard error of the mean (*n* = 5).

When virus responses of the CBSD-susceptible variety Albert were compared with that of the virus-resistant Namikonga (Figure 4), similar reaction patterns were found, although symptoms in Namikonga appeared delayed and remained very mild with distinct symptoms only occasionally found on single leaves. In later infection stages, virus concentrations dropped to almost undetectable levels (Figure 4B), but nevertheless, virus infections were maintained in this variety.

Finally, based on the early expression of symptoms and the rapid accumulation of virus RNA, CBSV-Mo83 was determined as the most pathogenic virus isolate among the CBSVs maintained in the reference virus collection at DSMZ. Consequently, CBSV-Mo83 was selected as inoculum for the initial virus screening.

### African Cassava Varieties and Breeding Lines Are Susceptible to CBSV-Mo83

In the virus bioassay, all African cassava lines became infected with CBSV-Mo83 and either were highly sensitive to the virus and responded with wilting followed by plant death or developed severe virus symptoms on leaves and stems. In our virus bioassays, the resistant varieties Namikonga and Kiroba (Maruthi et al., 2014; Nzuki et al., 2017) became infected and developed distinct CBSD symptoms; however, both varieties recovered from the disease at later infection stages to show only mild symptoms on newly developed leaves or no symptoms at all. Nevertheless, symptoms occasionally found on leaves confirmed that the virus was still present. The African varieties TZ-130 (NARO-CASS 1), KBH 2002/066 (Kipusa), KBH 2002/482 (Kizimbani), and KBH 2006/26, which in earlier studies were rated as CBSD resistant (Anjanappa et al., 2016; Kaweesi et al., 2016), also became readily infected with CBSV-Mo83. Under our infection conditions, those varieties responded with severe virus symptoms, wilting, and plant death (Supplementary Table 2).

### South American Cassava Varieties Show Differential Responses to Infections With CBSV-Mo83

**FIGURE 5:**
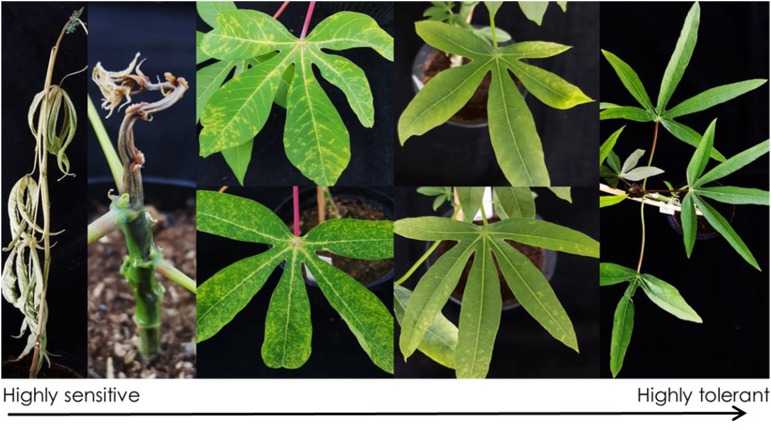
Symptoms from CBSV-Mo83 infections in cassava germplasm lines from South America. Highly sensitive varieties responded to virus infection with wilting and plant death 3–4 weeks after infection. Leaf symptoms range from severe vein chlorosis to mild mottling on leaves. Highly tolerant varieties did not show symptoms, and virus was not detectable.

Most of the South American cassava lines were susceptible to CBSV infection and to developing systemic symptoms ranging from vein clearing to wilting and plant death (Figure 5, 6C). Several cassava germplasm accessions from South America responded to CBSV infection with wilting of stems followed by plant death already 10–14 dag. Another group of plants developed systemic virus infections with severe symptoms on leaves and stems and a decline of the entire plant (Supplementary Table 2). In a third group of cassava plants, only mild leaf symptoms became visible approximately 14–21 dag, but the progression of the disease was much delayed (Supplementary Table 3). The cassava germplasm lines in these three groups were considered moderate to highly susceptible to CBSV-Mo83 and, thus, were excluded from further virus testing. Finally, after 5–8 months of symptom observation, only a small group of cassava accessions remained that survived the stringent virus testing conditions. Those cassava lines were free of symptoms with no virus detected in leaves or stem tissues.

The 238 cassava South American cassava lines included in the CBSV-Mo83 bioassay responded to virus infections, with symptoms ranging from wilting followed by plant death to absence of CBSV-Mo83 symptoms (Figure 5).

### South American Cassava Germplasm Bares Resistance Against CBSV-Mo83

Cassava varieties that remained virus free during the first screening phase were subjected to a second round of screening, during which several candidate accessions became virus infected. However, after two rounds of high stringency screening, 15 cassava lines remained free of virus symptoms, and there was no virus detected.

### CBSV-Mo83 Is Restricted to the Roots

**FIGURE 6:**
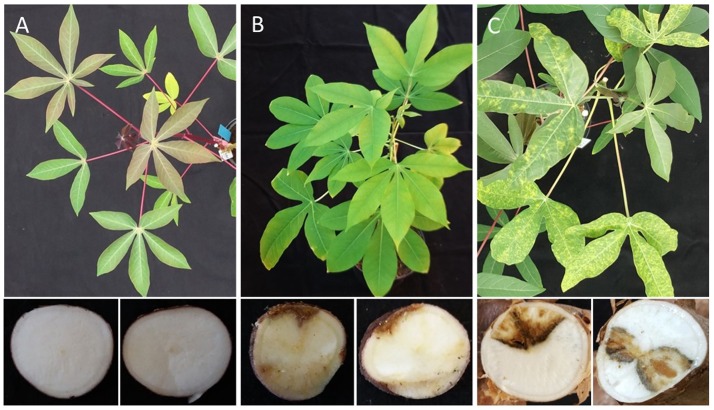
Differential response of cassava germplasm lines from South America to infections with CBSV-Mo83. Sensitive varieties **(C)** responded with symptoms on leaves and stems as well as marked root necrosis. Highly resistant varieties **(A)** do not become infected; resistant varieties **(B)** remain symptomless in aboveground plant parts and restrict the virus to the roots where necrosis symptoms can be observed.

At the end of the bioassay, after 5 to 8 months, when tuberous roots from the 15 apparently virus-free cassava lines from South America were examined, tubers from 8 cassava lines (DSC260, DSC261, DSC122, DSC248, DSC251, DSC199, DSC257, and DSC272) showed necrosis symptoms, and virus was detected by qRT-PCR (Table 1). Leaves and stems of those lines were free of symptoms, and also, CBSV-Mo83 was not detectable, confirming that virus infections were restricted to tuberous root tissues only (Figure 6B).

**Table 1:** Resistance against CBSV-Mo83 in South American cassava germplasm accessions.

Type of resistance	Nr.	DSMZ acronym	CIAT accession	Symptoms			qRT-PCR		
				Leaf	Stem	Root	Leaf	Stem	Root
No virus detected	1	DSC118	COL 40	–	–	–	–	–	–
	2	DSC167	COL 2182	–	–	–	–	–	–
	3	DSC196	ECU 41	–	–	–	–	–	–
	4	DSC250	PER 221	–	–	–	–	–	–
	5	DSC269	PER 556	–	–	–	–	–	–
	6	DSC120	COL 144	–	–	–	–	–	–
	7	DSC258	PER 333	–	–	–	–	–	–
Virus restricted to the roots	8	DSC122	COL 262	–	–	+	–	–	+
	9	DSC248	PER 206	–	–	+	–	–	+
	10	DSC251	PER 226	–	–	+	–	–	+
	11	DSC199	ECU 159	–	–	+	–	–	+
	12	DSC257	PER 315	–	–	+	–	–	+
	13	DSC260	PER 353	–	–	+	–	–	+
	14	DSC261	PER 368	–	–	+	–	–	+
	15	DSC272	PER 597	–	–	+	–	–	+

*qRT-PCT, quantitative reverse transcription PCR.*

### CBSV-Mo83 Infections Cannot Establish

CBSV-Mo83 infections did not establish in seven cassava lines: DSC167, DSC196, DSC250, DSC258, DSC120, DSC269, and DSC118. All inoculated plants were free of symptoms, and there was no virus detected in all organs and tissues tested (Figure 6A).

### Virus Resistance Also Holds Up Against Other U/CBSV Isolates

The virus isolates selected for the bioassays were from the three major phylogenetic clades: CBSV, CBSV-Tan70, and UCBSV (Winter et al., 2010; Ndunguru et al., 2015; Alicai et al., 2016; Mbewe et al., 2017), and differ in their aggressiveness to infect cassava (Figure 2, 3). When the resistant DSC167 and DSC118 were graft inoculated with axillary buds from plants infected with UCBSV-Ke125 and CBSV-Tan70, the cassava lines remained healthy. There were no symptoms of an evolving virus infection, and there was no virus detected in the plants.

### Virus Replication but Not Virus Movement Is Obstructed in the Cassava Line DSC167

**FIGURE 7:**
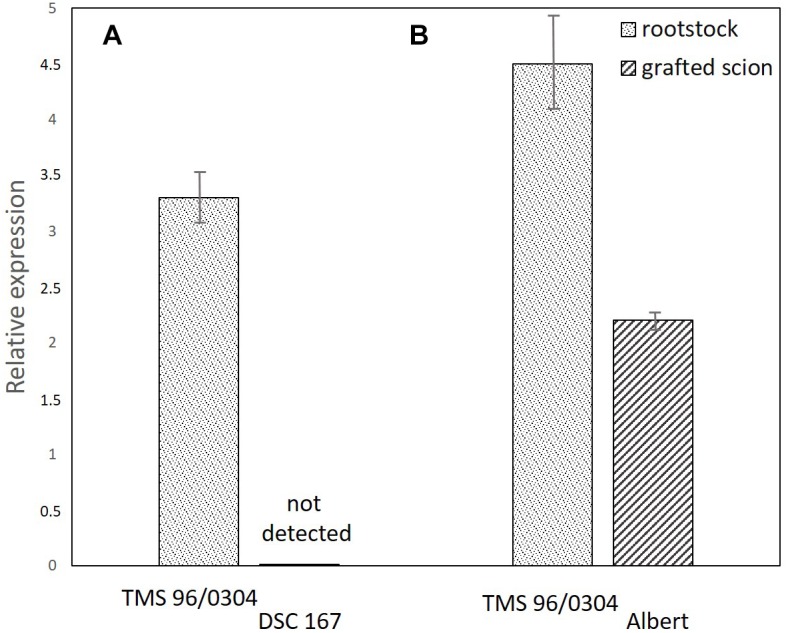
Quantification of CBSV-Mo83 in panel **(A)** cassava TMS-96/0304 rootstocks with growing branches of DSC167 (11 plants) in which no virus was detected and **(B)** cassava TMS-96/0304 rootstocks with growing branches of the sensitive variety Albert (six plants) with severe symptoms and high virus concentration. Virus expression values (*y* axis) were calculated relative to CBSV-Mo83 concentrations recorded in the sixth uppermost leaf of the variety TMS-96/0304.

To exclude that tissue incompatibility, necrosis, or other barriers would prevent translocation and movement of virus from infected buds into rootstocks, a further virus infection experiment was conducted with DSC167 and the variety Albert as susceptible controls. A chimeric TMS-96/0304 rootstock with a branched canopy, one branch from TMS-96/0304 and the other from DSC167, was infected by grafting CBSV-Mo83 buds to the susceptible rootstock. Virus symptoms became visible 10–14 dag on the leaves of TMS-96/0304 (11 of 12 grafted plants), and systemic virus infections with pronounced symptoms and high virus load developed in the following weeks. In the TMS-96/0304/Albert chimeric plants (six of six grafted plants), virus symptoms became visible on leaves of both branches, and a severe systemic disease developed. In contrast, the DSC167 branches of all chimeric plants remained symptomless throughout, and virus was undetected or below the qRT-PCR detection threshold in stem and leaf tissues taken from those branches (Figure 7). Cuttings taken from those DSC167 branches grew into healthy, symptom-free plants. When scions of the sensitive TMS-96/0304 were grafted onto those plants, all but one remained virus-free, thus confirming that CBSV-Mo83 was not maintained in the resistant DSC167. The only DSC167 offspring still carrying the infectious virus was a rooted cutting taken in close proximity to the graft junction. Hence, spillover virus may have been sufficient to infect the sensitive indicator line.

### CBSV Resistance in South American Cassava Germplasm

After two cycles of stringent virus screening and approximately 24 months of intense monitoring, 15 cassava lines with resistance against CBSV-Mo83 were identified in the germplasm accessions from South America (Table 1).

High resistance against CBSV-Mo83 was found in seven cassava lines, and those did not become infected with CBSV-Mo83. Additional virus bioassays with CBSV and UCBSV isolates, CBSV-UG65, CBSV-Tan70, and UCBSV-Ke125 provided evidence that virus resistance was also effective against diverse viruses. Virus testing with other cassava accessions are not yet finalized to conclude on their resistance spectrum.

## Discussion

Resistance against the viruses causing cassava mosaic and CBSDs is key to cassava cultivation in Africa and, thus, is a vital element of all breeding programs aimed at genetic improvement of this important food crop. Breeding for resistance against Cassava mosaic viruses, causing the most widespread cassava disease on the African continent (Patil and Fauquet, 2009), has been very successful, and to date, cassava varieties with broad-spectrum resistance against African and East African cassava mosaic species and strains are widely grown (Egesi et al., 2007; Dixon and Ssemakula, 2008; Lokko et al., 2009; Dixon et al., 2010; Rabbi et al., 2014).

Since the first report on the re-emergence of CBSD from Uganda in 2007 (Alicai et al., 2007), efforts with increasing intensity have been made to identify sources of resistance also against the viruses causing the brown streak disease (Kawuki et al., 2016). However, in contrast to CMD, the level of resistance against CBSVs found in some African cassava cultivars (Kawuki et al., 2016; Mukiibi et al., 2019) cannot be considered sufficient to reach comprehensive, broad-spectrum, and durable protection against the viruses. Indeed, there are less sensitive varieties with reduced virus titers and fewer root necrosis symptoms (Kaweesi et al., 2014; Kawuki et al., 2016; Kayondo et al., 2018; Tumwegamire et al., 2018), but all varieties are susceptible to the viruses and eventually succumb to the disease. One main source of resistance against viruses causing CBSD is Namikonga (Kaweesi et al., 2014; Kawuki et al., 2016; Masumba et al., 2017), a variety closely related to an interspecific hybrid with *M. glaziovii* (Hybrid 46106/27, Kaleso), which was developed during the Amani program (Jennings, 1957; Hillocks and Jennings, 2003). Namikonga, while sensitive to CMD, reacts to U/CBSV infections with only mild disease symptoms, reduced virus titers, and low incidence of root necrosis (Kaweesi et al., 2014). It is widely used in the current African breeding programs, and this emphasizes the limited availability of CBSD resistance in African cassava. This deficit provided the motivation for us to search for U/CBSV resistance in South American germplasm. The fact that CBSD resistance sources used in the early breeding programs were wild and domesticated Cassava ancestors from South America led to our belief that further resistance may exist in the untapped genetic diversity of cassava, which is preserved in the germplasm collection maintained at CIAT.

The screening approaches and selection criteria for which we have decided aimed at identifying cassava genotypes that would not become infected with any U/CBSV isolate. We reasoned that a once established infection with UCBSV may eventually lead to root necrosis in all cassava varieties, and even those considered as virus resistant (e.g., Namikonga) would eventually show root symptoms. Although incidence and severity of root symptoms can be low in those varieties, propagation of cuttings from virus-infected plants (secondary infection) eventually leads to an increase of root symptoms in successive growing cycles and to more severe impact. Thus, in our screening protocol, a cassava variety developing virus symptoms was eliminated from further testing, notwithstanding symptom severity, onset of infection, and virus titer.

The challenge of cassava virus resistance testing under laboratory (glasshouse) conditions was to establish an efficient virus infection method that would allow the use of only a few plants of each accession for infection experiments in the first high-throughput screening. We therefore increased the virus infection efficiency to more than 90% by using two axillary buds from virus-infected source plants to infect cassava by grafting. Indeed, this treatment did not only guarantee that almost all plants became infected but also triggered early plant responses because of the high virus inoculum delivered.

Considerable differences in accumulation of CBSV and UCBSV in cassava were reported (Kaweesi et al., 2014; Ogwok et al., 2016), and virus concentrations were measured for CBSV that were 10- to 1,000-fold higher than UCBSV. This can be taken as proof for the aggressiveness of CBSV viruses and their ability to rapidly replicate and invade their hosts. In contrast, the low UCBSV concentrations measured in cassava may indicate for delayed infection processes, although the diseases caused by both viruses in cassava including the incidence and severity of root symptoms are very similar. Indeed, the cassava infections caused by the U/CBSV isolates we have compared differed particularly in the early stages of the disease. CBSV isolates reached higher rates of replication and caused more severe symptoms in cassava (Figure 2). Because in systemic infections, virus replication is linked with host invasion, it can be speculated that aggressive virus isolates reach cassava roots earlier and consequently cause more severe root necrosis symptoms than virus isolates with limited replication potential and delayed systemic movement. When root symptoms are taken as a measure of virus resistance/tolerance in cassava, it needs to be considered that the extent of root necrosis is correlated with the pathogenicity of a virus isolate and the length of a persisting infection. UCBSV infections in cassava can progress very slowly and are then associated with low rates of virus replication. In our study, it could take more than 6 months for some cassava lines to show symptoms, and virus was not detected before. Thus observation periods of 16 weeks only (Anjanappa et al., 2016) may have not been sufficient to assess the resistance status of the cassava line KBH 2006/26, which then failed in our tests. In addition, our comparative study (Figure 2) also showed that mild CBSV isolates (CBSV-Tan70) that exist in symptom development and virus accumulation resemble UCBSV. This emphasizes that defined virus (reference) isolates shall be used in virus resistance studies to reach comparable and reproducible results since obscure sources of partially or uncharacterized viruses only add doubts about the accuracy of the experimental evidence provided.

The stringent conditions we used to infect South American and African cassava varieties, i.e., the use of an aggressive virus in a highly effective infection process, allowed us to eliminate already in the first cycle of virus screening (Figure 1) more than 90% of susceptible plants with only few ambiguous cases left for verification in a second selection cycle. We selected for absence of symptoms and virus in aboveground plant parts, and those South American cassava varieties finally passing the screen were free of virus symptoms and tested negative for virus presence in qRT-PCR. Only in the final, destructive test after 6–8 months of observation, it became evident that among the cassava lines considered as free of virus, there were accessions having symptoms on tuberous roots and a CBSV infection that was localized to root tissues only. A similar observation was made with citrus tristeza virus (CTV) isolates infecting citrus and other *Rutacea* that are commonly used as rootstocks (Harper et al., 2014). In CTV-resistant hosts, virus invasion of the roots was comparable to susceptible species, but CTV remained localized in root tissues and also was not detected in shoots. This means that conditions for virus invasion of roots and shoots differ, and specific constraints may exist for viruses to infect shoot tissues. To this date, nothing is known about specific functions of U/CBSV genes in cell-to-cell and/or long-distance movement and their interaction with host factors. Thus, the underlying mechanism regulating root tropism of CBSV in cassava lines remains to be elucidated.

The failure of CBSV-Mo83 to establish in resistant cassava varieties like DSC167 may indicate an incompatibility to sustain virus replication because (co-evolved) genes necessary for precise interaction with host proteins or structures are missing. On the other hand, the resistance phenotype may be the lack of replication or because the phloem cells represent an impermeable physical barrier to prevent passage of the virus to mesophyll cells for replication.

Our infection experiments were rigorous and reproducible to provide strong evidence for cassava resistance (Table 1). However, it has to be emphasized that all observations require validation under field situations, and this is particularly important for the assessment of symptoms on root tissues. Root development is greatly impeded when cassava is grown under glasshouse conditions, and thus, uncertainty exists whether for some varieties sufficient root tissues were investigated to unequivocally assign a particular genotype to the group of highly resistant varieties.

The screening strategy we chose was based on the reasoning that resistance against the most aggressive virus isolate, CBSV-Mo83, would not only be most efficient to eliminate susceptible lines but may also lead to the identification of broad-spectrum resistance against other U/CBSV isolates. Indeed, in an earlier study, we identified UCBSV resistance in cassava varieties that were otherwise susceptible against CBSV (Winter et al., 2010). The results of the bioassays with other viruses concluded for cassava DSC167 and DSC118 show that varieties with broad resistance spectrum against CBSV and UCBSV isolates exist. While these results are highly encouraging, care must be taken not to deduce from these findings any conclusions about others, and thus, it cannot be inferred that resistance against a highly pathogenic virus isolate would also hold against less virulent viruses.

In field situations, scoring for U/CBSV resistance is problematic because of the uncertainties about (1) the presence and identity of (a) particular virus (es), (2) the infection pressure (whitefly population size), and (3) the time point of infection. These inaccuracies have a severe impact on the entire cassava breeding process and prolong the selection and evaluation to 3–4 years (Kawuki et al., 2016). The current practice of field evaluations for cassava resistance responses generally take CBSD symptoms on leaves and roots into account. The diversity of virus genomes (Ndunguru et al., 2015; Alicai et al., 2016), the presence of viruses in single and mixed virus infections, and the strikingly different host responses (Winter et al., 2010; Ogwok et al., 2016), however, are not adequately addressed. Hence, we consider it important to increase the precision and efficiency of U/CBSV resistance breeding by taking control of all elements of the process, from the time point of infection with a known virus isolate to using the right tissues and time points for resistance scoring. The study we present here can provide a directive for improving the currently followed resistance selection process.

In this work, we present South American cassava breeding lines that are resistant against CBSV and other viruses encompassing the diversity of viruses causing CBSD. Evaluation of some of the lines grown at several sites in East Africa confirmed that the resistance against both viruses, CBSV and UCBSV, was maintained. While the appraisal is still ongoing, we are confident that these new cassava materials can provide the genetic sources needed to make substantial progress in cassava breeding for CBSD resistance. Finally, this work highlights the invaluable contribution of germplasm collections to supply the genetic resources for the improvement of our crops.

## Author Contributions

SW conceived the project and designed the experiments. SS and BF carried out the experiments and generated the data. SS, BF and SW analyzed the data. SS and SW wrote the manuscript. All authors contributed critically to the manuscript and agreed to the final content.

## Conflict of Interest Statement

The authors declare that the research was conducted in the absence of any commercial or financial relationships that could be construed as a potential conflict of interest.

## Footnotes

1 https://talk.ictvonline.org/files/master-species-lists/m/msl/8266

2 https://cgspace.cgiar.org/handle/10568/43737
