# Supplementary material for: Resistance Against Cassava Brown Streak Viruses From Africa in Cassava Germplasm From South America
Source: Front Plant Sci. 2019 May 10;10:567. doi: 10.3389/fpls.2019.00567 (PMC6523400; doi:10.3389/fpls.2019.00567)
Supplement: Supplementary file 1 [file Table_1.DOCX]

Table 1. Primers and probes used for U/ CBSV detection and quantification by RT-qPCR

| **primer description** | **primer name** | **primer sequence (5'-3')** | **Reference** |
| --- | --- | --- | --- |
| Uganda cassava brown streak virus | UCBSV forward | GATYAARAAGACITTCAAGCCTCCAAA | Adams et al., 2013 |
|  | UCBSV reverse | AATTACATCAGGRGTTAGRTTRTCCCTT | Adams et al., 2013 |
|  | UCBSV probe | FAM- TCAGCTTACATTTGGATTCCACGCTCTCA- TAMRA | Adams et al., 2013 |
| Cassava brown streak virus | CBSV forward | GCCAACTARAACTCGAAGTCCATT | Adams et al., 2013 |
|  | CBSV reverse | TTCAGTTGTTTAAGCAGTTCGTTCA | Adams et al., 2013 |
|  | CBSV probe | FAM- AGTCAAGGAGGCTTCGTGCYCCTC -BHQ1 | Adams et al., 2013 |
| Cytochrome oxidase | COX forward | CGTCGCATTCCAGATTATCCA | Kaweesi et al., 2014 |
|  | COX reverse | CAACTACGGATATATAAGRRCCRRAACTG | Kaweesi et al., 2014 |
|  | COX probe | FAM-AGGGCATTCCATCCAGCGTAAGCA-TAMRA | Kaweesi et al., 2014 |
| Acyl-CoA-binding protein | cassava4.1_010236 forward | GATGCCATTCATGCCTTTG | Hu et al., 2016 |
|  | cassava4.1_010236 reverse | TCCGACCCTCGCTATCTTT | Hu et al., 2016 |

Table 2: Symptoms of CBSV-Mo83 infections in African cassava lines

| **No** | **DSMZ acronym** | **African accession** | **symptom severity** |
| --- | --- | --- | --- |
| **1** | DSC 334 | 72-TME14 | wilting, followed by plant death |
| **2** | DSC 335 | Nase 14 | wilting, followed by plant death |
| **3** | DSC 298 | Nase 4 | wilting, followed by plant death |
| **4** | DSC 308 | Shibe | wilting, followed by plant death |
| **5** | DSC 301 | Yizaso | wilting, followed by plant death |
| **6** | DSC 323 | Eyope | wilting, followed by plant death |
| **7** | DSC 314 | F10-30-R2 | wilting, followed by plant death |
| **8** | DSC 327 | KBH 2002/066 (Kipusa) | wilting, followed by plant death |
| **9** | DSC 321 | Sauti | wilting, followed by plant death |
| **10** | DSC 333 | Nam 130 | wilting, followed by plant death |
| **11** | DSC 330 | Kizimbani | wilting, followed by plant death |
| **12** | DSC 336 | TME204 | wilting, followed by plant death |
| **13** | DSC 362 | 2001/1661 | wilting, followed by plant death |
| **14** | DSC 322 | Oekhumelela | wilting, followed by plant death |
| **15** | DSC 319 | Soganja | wilting, followed by plant death |
| **16** | DSC 331 | KBH 2006/26 (Mkuranga) | wilting, followed by plant death |
| **17** | DSC 307 | Nase 18 | wilting, followed by plant death |
| **18** | DSC 310 | TZ 130 | severe symptoms on leaves and stems |
| **19** | DSC 318 | CHO5/203 | severe symptoms on leaves and stems |
| **20** | DSC 315 | Kibandemeno | severe symptoms on leaves and stems |
| **21** | DSC 317 | Mbundumali | severe symptoms on leaves and stems |
| **22** | DSC 299 | Nase 13 | severe symptoms on leaves and stems |
| **23** | DSC 332 | Nase 3* | severe symptoms on leaves and stems |
| **24** | DSC 324 | Nziva | severe symptoms on leaves and stems |
| **25** | DSC 326 | Orera | severe symptoms on leaves and stems |
| **26** | DSC 328 | Pwani | severe symptoms on leaves and stems |
| **27** | DSC 320 | Kalawe | severe symptoms on leaves and stems |
| **28** | DSC 311 | LM1/2008/363 | severe symptoms on leaves and stems |
| **29** | DSC 325 | Colicanana | severe symptoms on leaves and stems |
| **30** | DSC 50 | Nanchinyaya | severe symptoms on leaves and stems |
| **31** | DSC 33 | AR-40-6* | severe symptoms on leaves and stems |
| **32** | DSC 31 | Albert | severe symptoms on leaves and stems |
| **33** | DSC 27 | TMS 96/0304 | severe symptoms on leaves and stems |
| **34** | DSC 53 | TME117 | severe symptoms on leaves and stems |
| **35** | DSC 45 | Kiroba | moderate symptoms on leaves and stems, transient |
| **36** | DSC 51 | Namikonga | moderate symptoms on leaves and stems, transient |
| **37** | DSC 302 | F19 | moderate symptoms on leaves only |
| **38** | DSC 316 | Mkumbozi | moderate symptoms on leaves only |
| **39** | DSC 300 | TME14 | moderate symptoms on leaves only |
| **40** | DSC 329 | Mkumba | moderate symptoms on leaves only |
| **41** | DSC 364 | TMS 4(2)1425 | moderate symptoms on leaves only |
| **42** | DSC 305 | Rasta | stem symptoms, inconspicuous symptoms on leaves |

*Lines with South American origin

Table 3. Severity of symptom in South American cassava varieties infected with CBSV-Mo83

| **nr.** | **DSMZ acronym** | **CIAT accession** | **symptom severity** |
| --- | --- | --- | --- |
| **1** | DSC 74 | BRA 315 | wilting, followed by plant death |
| **2** | DSC 78 | BRA 400 | wilting, followed by plant death |
| **3** | DSC 82 | BRA 453 | wilting, followed by plant death |
| **4** | DSC 85 | BRA 472 | wilting, followed by plant death |
| **5** | DSC 96 | BRA 702 | wilting, followed by plant death |
| **6** | DSC 106 | BRA 881 | wilting, followed by plant death |
| **7** | DSC 110 | BRA 897 | wilting, followed by plant death |
| **8** | DSC 114 | BRA 924 | wilting, followed by plant death |
| **9** | DSC 119 | COL 87 | wilting, followed by plant death |
| **10** | DSC 127 | COL 337 | wilting, followed by plant death |
| **11** | DSC 128 | COL 346 | wilting, followed by plant death |
| **12** | DSC 129 | COL 451 | wilting, followed by plant death |
| **13** | DSC 145 | COL 1186A | wilting, followed by plant death |
| **14** | DSC 149 | COL 1466 | wilting, followed by plant death |
| **15** | DSC 151 | COL 1486 | wilting, followed by plant death |
| **16** | DSC 152 | COL 1516 | wilting, followed by plant death |
| **17** | DSC 170 | COL 2306 | wilting, followed by plant death |
| **18** | DSC 171 | COL 2315 | wilting, followed by plant death |
| **19** | DSC 175 | COL 2387 | wilting, followed by plant death |
| **20** | DSC 176 | COL 2426 | wilting, followed by plant death |
| **21** | DSC 183 | CUB 1 | wilting, followed by plant death |
| **22** | DSC 188 | CUB 51 | wilting, followed by plant death |
| **23** | DSC 192 | ECU 3 | wilting, followed by plant death |
| **24** | DSC 206 | GUA 41 | wilting, followed by plant death |
| **25** | DSC 209 | MAL 13 | wilting, followed by plant death |
| **26** | DSC 211 | MAL 27 | wilting, followed by plant death |
| **27** | DSC 212 | MAL 35 | wilting, followed by plant death |
| **28** | DSC 213 | MAL 50 | wilting, followed by plant death |
| **29** | DSC 214 | MAL 59 | wilting, followed by plant death |
| **30** | DSC 215 | MAL 60 | wilting, followed by plant death |
| **31** | DSC 217 | MEX 27 | wilting, followed by plant death |
| **32** | DSC 225 | NGA 2 | wilting, followed by plant death |
| **33** | DSC 230 | PAR 18 | wilting, followed by plant death |
| **34** | DSC 231 | PAR 23 | wilting, followed by plant death |
| **35** | DSC 238 | PAR 69 | wilting, followed by plant death |
| **36** | DSC 240 | PAR 98 | wilting, followed by plant death |
| **37** | DSC 252 | PER 234 | wilting, followed by plant death |
| **38** | DSC 273 | PER 612 | wilting, followed by plant death |
| **39** | DSC 60 | ARG 2 | severe symptoms on leaves and stems |
| **40** | DSC 61 | BOL 1 | severe symptoms on leaves and stems |
| **41** | DSC 62 | BRA 18 | severe symptoms on leaves and stems |
| **42** | DSC 64 | BRA 77 | severe symptoms on leaves and stems |
| **43** | DSC 65 | BRA 110 | severe symptoms on leaves and stems |
| **44** | DSC 66 | BRA 125 | severe symptoms on leaves and stems |
| **45** | DSC 67 | BRA 130 | severe symptoms on leaves and stems |
| **46** | DSC 69 | BRA 165 | severe symptoms on leaves and stems |
| **47** | DSC 72 | BRA 299 | severe symptoms on leaves and stems |
| **48** | DSC 73 | BRA 311 | severe symptoms on leaves and stems |
| **49** | DSC 75 | BRA 328 | severe symptoms on leaves and stems |
| **50** | DSC 81 | BRA 416 | severe symptoms on leaves and stems |
| **51** | DSC 83 | BRA 461 | severe symptoms on leaves and stems |
| **52** | DSC 84 | BRA 467 | severe symptoms on leaves and stems |
| **53** | DSC 86 | BRA 475 | severe symptoms on leaves and stems |
| **54** | DSC 87 | BRA 507 | severe symptoms on leaves and stems |
| **55** | DSC 89 | BRA 590 | severe symptoms on leaves and stems |
| **56** | DSC 90 | BRA 658 | severe symptoms on leaves and stems |
| **57** | DSC 92 | BRA 692 | severe symptoms on leaves and stems |
| **58** | DSC 93 | BRA 697 | severe symptoms on leaves and stems |
| **59** | DSC 94 | BRA 698 | severe symptoms on leaves and stems |
| **60** | DSC 95 | BRA 699 | severe symptoms on leaves and stems |
| **61** | DSC 98 | BRA 730 | severe symptoms on leaves and stems |
| **62** | DSC 100 | BRA 781 | severe symptoms on leaves and stems |
| **63** | DSC 103 | BRA 847 | severe symptoms on leaves and stems |
| **64** | DSC 104 | BRA 852 | severe symptoms on leaves and stems |
| **65** | DSC 105 | BRA 854 | severe symptoms on leaves and stems |
| **66** | DSC 107 | BRA 887 | severe symptoms on leaves and stems |
| **67** | DSC 113 | BRA 915 | severe symptoms on leaves and stems |
| **68** | DSC 115 | BRA 931 | severe symptoms on leaves and stems |
| **69** | DSC 117 | COL 32 | severe symptoms on leaves and stems |
| **70** | DSC 121 | COL 226B | severe symptoms on leaves and stems |
| **71** | DSC 124 | COL 304 | severe symptoms on leaves and stems |
| **72** | DSC 125 | COL 306 | severe symptoms on leaves and stems |
| **73** | DSC 131 | COL 511 | severe symptoms on leaves and stems |
| **74** | DSC 132 | COL 634 | severe symptoms on leaves and stems |
| **75** | DSC 133 | COL 638 | severe symptoms on leaves and stems |
| **76** | DSC 134 | COL 809B | severe symptoms on leaves and stems |
| **77** | DSC 136 | COL 890 | severe symptoms on leaves and stems |
| **78** | DSC 137 | COL 955 | severe symptoms on leaves and stems |
| **79** | DSC 138 | COL 965 | severe symptoms on leaves and stems |
| **80** | DSC 144 | COL 1137 | severe symptoms on leaves and stems |
| **81** | DSC 147 | COL 1398 | severe symptoms on leaves and stems |
| **82** | DSC 148 | COL 1413 | severe symptoms on leaves and stems |
| **83** | DSC 154 | COL 1535 | severe symptoms on leaves and stems |
| **84** | DSC 156 | COL 1722 | severe symptoms on leaves and stems |
| **85** | DSC 157 | COL 1786 | severe symptoms on leaves and stems |
| **86** | DSC 159 | COL 1890 | severe symptoms on leaves and stems |
| **87** | DSC 161 | COL 2019 | severe symptoms on leaves and stems |
| **88** | DSC 166 | COL 2177 | severe symptoms on leaves and stems |
| **89** | DSC 168 | COL 2199 | severe symptoms on leaves and stems |
| **90** | DSC 169 | COL 2212 | severe symptoms on leaves and stems |
| **91** | DSC 173 | COL 2331 | severe symptoms on leaves and stems |
| **92** | DSC 174 | COL 2353 | severe symptoms on leaves and stems |
| **93** | DSC 177 | CR 18 | severe symptoms on leaves and stems |
| **94** | DSC 178 | CR 19 | severe symptoms on leaves and stems |
| **95** | DSC 181 | CR 101 | severe symptoms on leaves and stems |
| **96** | DSC 182 | CR 133 | severe symptoms on leaves and stems |
| **97** | DSC 185 | CUB 36 | severe symptoms on leaves and stems |
| **98** | DSC 186 | CUB 40 | severe symptoms on leaves and stems |
| **99** | DSC 187 | CUB 46 | severe symptoms on leaves and stems |
| **100** | DSC 189 | CUB 53 | severe symptoms on leaves and stems |
| **101** | DSC 190 | CUB 58 | severe symptoms on leaves and stems |
| **102** | DSC 195 | ECU 33 | severe symptoms on leaves and stems |
| **103** | DSC 197 | ECU 82 | severe symptoms on leaves and stems |
| **104** | DSC 198 | ECU 144 | severe symptoms on leaves and stems |
| **105** | DSC 205 | GUA 32 | severe symptoms on leaves and stems |
| **106** | DSC 207 | IND 11 | severe symptoms on leaves and stems |
| **107** | DSC 216 | MEX 2 | severe symptoms on leaves and stems |
| **108** | DSC 218 | MEX 45 | severe symptoms on leaves and stems |
| **109** | DSC 219 | MEX 49 | severe symptoms on leaves and stems |
| **110** | DSC 220 | MEX 55 | severe symptoms on leaves and stems |
| **111** | DSC 222 | MEX 83 | severe symptoms on leaves and stems |
| **112** | DSC 226 | NGA 16 | severe symptoms on leaves and stems |
| **113** | DSC 229 | PAR 15 | severe symptoms on leaves and stems |
| **114** | DSC 233 | PAR 32 | severe symptoms on leaves and stems |
| **115** | DSC 234 | PAR 35 | severe symptoms on leaves and stems |
| **116** | DSC 235 | PAR 38 | severe symptoms on leaves and stems |
| **117** | DSC 236 | PAR 41 | severe symptoms on leaves and stems |
| **118** | DSC 237 | PAR 57 | severe symptoms on leaves and stems |
| **119** | DSC 244 | PAR 163 | severe symptoms on leaves and stems |
| **120** | DSC 246 | PER 192 | severe symptoms on leaves and stems |
| **121** | DSC 249 | PER 209 | severe symptoms on leaves and stems |
| **122** | DSC 253 | PER 283 | severe symptoms on leaves and stems |
| **123** | DSC 254 | PER 290 | severe symptoms on leaves and stems |
| **124** | DSC 256 | PER 295 | severe symptoms on leaves and stems |
| **125** | DSC 259 | PER 349 | severe symptoms on leaves and stems |
| **126** | DSC 266 | PER 484 | severe symptoms on leaves and stems |
| **127** | DSC 270 | PER 584 | severe symptoms on leaves and stems |
| **128** | DSC 277 | PTR 55 | severe symptoms on leaves and stems |
| **129** | DSC 278 | PTR 102 | severe symptoms on leaves and stems |
| **130** | DSC 279 | TAI 1 | severe symptoms on leaves and stems |
| **131** | DSC 280 | VEN 25 | severe symptoms on leaves and stems |
| **132** | DSC 283 | VEN 90 | severe symptoms on leaves and stems |
| **133** | DSC 287 | VEN 164 | severe symptoms on leaves and stems |
| **134** | DSC 288 | VEN 167 | severe symptoms on leaves and stems |
| **135** | DSC 292 | VEN 284A | severe symptoms on leaves and stems |
| **136** | DSC 294 | VEN 297A | severe symptoms on leaves and stems |
| **137** | DSC 295 | VEN 298 | severe symptoms on leaves and stems |
| **138** | DSC 68 | BRA 162 | moderate symptoms on leaves only |
| **139** | DSC 70 | BRA 242 | moderate symptoms on leaves only |
| **140** | DSC 71 | BRA 243 | moderate symptoms on leaves only |
| **141** | DSC 76 | BRA 335 | moderate symptoms on leaves only |
| **142** | DSC 77 | BRA 359 | moderate symptoms on leaves only |
| **143** | DSC 79 | BRA 403 | moderate symptoms on leaves only |
| **144** | DSC 80 | BRA 405 | moderate symptoms on leaves only |
| **145** | DSC 91 | BRA 675 | moderate symptoms on leaves only |
| **146** | DSC 97 | BRA 712 | moderate symptoms on leaves only |
| **147** | DSC 99 | BRA 759 | moderate symptoms on leaves only |
| **148** | DSC 101 | BRA 792 | moderate symptoms on leaves only |
| **149** | DSC 102 | BRA 819 | moderate symptoms on leaves only |
| **150** | DSC 108 | BRA 890 | moderate symptoms on leaves only |
| **151** | DSC 111 | BRA 900 | moderate symptoms on leaves only |
| **152** | DSC 112 | BRA 903 | moderate symptoms on leaves only |
| **153** | DSC 116 | COL 22 | moderate symptoms on leaves only |
| **154** | DSC 123 | COL 299 | moderate symptoms on leaves only |
| **155** | DSC 130 | COL 490 | moderate symptoms on leaves only |
| **156** | DSC 135 | COL 856 | moderate symptoms on leaves only |
| **157** | DSC 139 | COL 976 | moderate symptoms on leaves only |
| **158** | DSC 140 | COL 1055 | moderate symptoms on leaves only |
| **159** | DSC 143 | COL 1108 | moderate symptoms on leaves only |
| **160** | DSC 146 | COL 1389 | moderate symptoms on leaves only |
| **161** | DSC 150 | COL 1467 | moderate symptoms on leaves only |
| **162** | DSC 155 | COL 1702 | moderate symptoms on leaves only |
| **163** | DSC 160 | COL 1999 | moderate symptoms on leaves only |
| **164** | DSC 162 | COL 2025 | moderate symptoms on leaves only |
| **165** | DSC 163 | COL 2061 | moderate symptoms on leaves only |
| **166** | DSC 172 | COL 2318 | moderate symptoms on leaves only |
| **167** | DSC 179 | CR 63 | moderate symptoms on leaves only |
| **168** | DSC 184 | CUB 32 | moderate symptoms on leaves only |
| **169** | DSC 191 | DOM 5 | moderate symptoms on leaves only |
| **170** | DSC 194 | ECU 23 | moderate symptoms on leaves only |
| **171** | DSC 200 | ECU 165 | moderate symptoms on leaves only |
| **172** | DSC 201 | ECU 166 | moderate symptoms on leaves only |
| **173** | DSC 202 | ECU 183 | moderate symptoms on leaves only |
| **174** | DSC 203 | FJI 6 | moderate symptoms on leaves only |
| **175** | DSC 204 | GUA 12 | moderate symptoms on leaves only |
| **176** | DSC 208 | MAL 2 | moderate symptoms on leaves only |
| **177** | DSC 221 | MEX 80 | moderate symptoms on leaves only |
| **178** | DSC 223 | MEX 95 | moderate symptoms on leaves only |
| **179** | DSC 239 | PAR 71 | moderate symptoms on leaves only |
| **180** | DSC 241 | PAR 104 | moderate symptoms on leaves only |
| **181** | DSC 243 | PAR 156 | moderate symptoms on leaves only |
| **182** | DSC 247 | PER 205 | moderate symptoms on leaves only |
| **183** | DSC 255 | PER 292 | moderate symptoms on leaves only |
| **184** | DSC 262 | PER 370 | moderate symptoms on leaves only |
| **185** | DSC 263 | PER 431 | moderate symptoms on leaves only |
| **186** | DSC 264 | PER 438 | moderate symptoms on leaves only |
| **187** | DSC 267 | PER 489 | moderate symptoms on leaves only |
| **188** | DSC 274 | PER 613 | moderate symptoms on leaves only |
| **189** | DSC 284 | VEN 130 | moderate symptoms on leaves only |
| **190** | DSC 286 | VEN 151 | moderate symptoms on leaves only |
| **191** | DSC 289 | VEN 174 | moderate symptoms on leaves only |
| **192** | DSC 290 | VEN 210 | moderate symptoms on leaves only |
| **193** | DSC 291 | VEN 244 | moderate symptoms on leaves only |
| **194** | DSC 293 | VEN 284B | moderate symptoms on leaves only |
| **195** | DSC 297 | VEN 321 | moderate symptoms on leaves only |
| **196** | DSC 63 | BRA 71 | stems symptoms, very mild, inconspicuous symptoms on leaves only |
| **197** | DSC 88 | BRA 542 | stems symptoms, very mild, inconspicuous symptoms on leaves only |
| **198** | DSC 109 | BRA 894 | stems symptoms, very mild, inconspicuous symptoms on leaves only |
| **199** | DSC 126 | COL 317 | stems symptoms, very mild, inconspicuous symptoms on leaves only |
| **200** | DSC 141 | COL 1084B | stems symptoms, very mild, inconspicuous symptoms on leaves only |
| **201** | DSC 153 | COL 1517 | stems symptoms, very mild, inconspicuous symptoms on leaves only |
| **202** | DSC 158 | COL 1805 | stems symptoms, very mild, inconspicuous symptoms on leaves only |
| **203** | DSC 180 | CR 77 | stems symptoms, very mild, inconspicuous symptoms on leaves only |
| **204** | DSC 193 | ECU 10 | stems symptoms, very mild, inconspicuous symptoms on leaves only |
| **205** | DSC 210 | MAL 24 | stems symptoms, very mild, inconspicuous symptoms on leaves only |
| **206** | DSC 224 | NGA 1 | stems symptoms, very mild, inconspicuous symptoms on leaves only |
| **207** | DSC 227 | PAN 7 | stems symptoms, very mild, inconspicuous symptoms on leaves only |
| **208** | DSC 228 | PAR 2 | stems symptoms, very mild, inconspicuous symptoms on leaves only |
| **209** | DSC 232 | PAR 25 | stems symptoms, very mild, inconspicuous symptoms on leaves only |
| **210** | DSC 242 | PAR 135 | stems symptoms, very mild, inconspicuous symptoms on leaves only |
| **211** | DSC 245 | PAR 193 | stems symptoms, very mild, inconspicuous symptoms on leaves only |
| **212** | DSC 265 | PER 449 | stems symptoms, very mild, inconspicuous symptoms on leaves only |
| **213** | DSC 268 | PER 503 | stems symptoms, very mild, inconspicuous symptoms on leaves only |
| **214** | DSC 271 | PER 589 | stems symptoms, very mild, inconspicuous symptoms on leaves only |
| **215** | DSC 275 | PTR 1 | stems symptoms, very mild, inconspicuous symptoms on leaves only |
| **216** | DSC 276 | PTR 8 | stems symptoms, very mild, inconspicuous symptoms on leaves only |
| **217** | DSC 281 | VEN 47 | stems symptoms, very mild, inconspicuous symptoms on leaves only |
| **218** | DSC 282 | VEN 69 | stems symptoms, very mild, inconspicuous symptoms on leaves only |
| **219** | DSC 285 | VEN 134 | stems symptoms, very mild, inconspicuous symptoms on leaves only |
| **220** | DSC 296 | VEN 309 | stems symptoms, very mild, inconspicuous symptoms on leaves only |
| **221** | DSC 164 | COL 2131 | stems symptoms, very mild, inconspicuous symptoms on leaves only |
| **222** | DSC 165 | COL 2173 | stems symptoms, very mild, inconspicuous symptoms on leaves only |
| **223** | DSC 118 | COL 40 | no symptoms on leaves and stems |
| **224** | DSC 120 | COL 144 | no symptoms on leaves and stems |
| **225** | DSC 122 | COL 262 | no symptoms on leaves and stems |
| **226** | DSC 167 | COL 2182 | no symptoms on leaves and stems |
| **227** | DSC 196 | ECU 41 | no symptoms on leaves and stems |
| **228** | DSC 199 | ECU 159 | no symptoms on leaves and stems |
| **229** | DSC 248 | PER 206 | no symptoms on leaves and stems |
| **230** | DSC 250 | PER 221 | no symptoms on leaves and stems |
| **231** | DSC 251 | PER 226 | no symptoms on leaves and stems |
| **232** | DSC 257 | PER 315 | no symptoms on leaves and stems |
| **233** | DSC 258 | PER 333 | no symptoms on leaves and stems |
| **234** | DSC 260 | PER 353 | no symptoms on leaves and stems |
| **235** | DSC 261 | PER 368 | no symptoms on leaves and stems |
| **236** | DSC 269 | PER 556 | no symptoms on leaves and stems |
| **237** | DSC 272 | PER 597 | no symptoms on leaves and stems |
| **238** | DSC 142 | COL 1107 | no symptoms on leaves and stems |
